# Factors influencing the integration of evidence-based task-strengthening strategies for hypertension control within HIV clinics in Nigeria

**DOI:** 10.1186/s43058-022-00289-z

**Published:** 2022-04-15

**Authors:** Juliet Iwelunmor, Oliver Ezechi, Chisom Obiezu-Umeh, David Oladele, Ucheoma Nwaozuru, Angela Aifah, Joyce Gyamfi, Titilola Gbajabiamila, Adesola Z. Musa, Deborah Onakomaiya, Ashlin Rakhra, Hu Jiyuan, Oluwatosin Odubela, Ifeoma Idigbe, Alexis Engelhart, Bamidele O. Tayo, Gbenga Ogedegbe

**Affiliations:** 1 College for Public Health and Social Justice, Saint Louis University, 3545 Lafayette Avenue, St. Louis, MO 63104 USA; 2 Nigerian Institute of Medical Research, Lagos, Nigeria; 3 Department of Population Health|, New York University School of Medicine, New York, NY USA; 4 Department of Preventive Medicine and Epidemiology, Loyola University Chicago, Stritch School of Medicine, Maywood, IL USA

**Keywords:** Hypertension control, HIV clinics, Implementation Climate, Nigeria

## Abstract

**Background:**

Evidence-based task-strengthening strategies for hypertension (HTN) control (TASSH) are not readily available for patients living with HIV in sub-Saharan Africa where the dual burden of HTN and HIV remains high. We are conducting a cluster randomized controlled trial comparing the effectiveness of practice facilitation versus a self-directed control (i.e., receipt of TASSH with no practice facilitation) in reducing blood pressure and increasing the adoption of task-strengthening strategies for HTN control within HIV clinics in Nigeria. Prior to implementing the trial, we conducted formative research to identify factors that may influence the integration of TASSH within HIV clinics in Nigeria.

**Methods:**

This mixed-methods study was conducted with purposively selected healthcare providers at 29 HIV clinics, followed by a 1-day stakeholder meeting with 19 representatives of HIV clinics. We collected quantitative practice assessment data using two instruments: (a) an adapted Service Availability and Readiness Assessment (SARA) tool to assess the capacity of the clinic to manage NCDs and (b) Implementation Climate Scale to assess the degree to which there is a strategic organizational climate supportive of the evidence-based practice implementation. The quantitative data were analyzed using descriptive statistics and measures of scale reliability. We also used the Consolidated Framework for Implementation Research (CFIR), to thematically analyze qualitative data generated and relevant to the aims of this study.

**Results:**

Across the 29 clinics surveyed, the focus on TASSH (mean=1.77 (SD=0.59)) and educational support (mean=1.32 (SD=0.68)) subscales demonstrated the highest mean score, with good–excellent internal consistency reliability (Cronbach’s alphas ranging from 0.84 to 0.96). Within the five CFIR domains explored, the major facilitators of the intervention included relative advantage of TASSH compared to current practice, compatibility with clinic organizational structures, support of patients’ needs, and intervention alignment with national guidelines. Barriers included the perceived complexity of TASSH, weak referral network and patient tracking mechanism within the clinics, and limited resources and diagnostic equipment for HTN.

**Conclusion:**

Optimizing healthcare workers’ implementation of evidence-based TASSH within HIV clinics requires attention to both the implementation climate and contextual factors likely to influence adoption and long-term sustainability. These findings have implications for the development of effective practice facilitation strategies to further improve the delivery and integration of TASSH within HIV clinics in Nigeria.

**Trial registration:**

ClinicalTrials.gov, NCT04704336

**Supplementary Information:**

The online version contains supplementary material available at 10.1186/s43058-022-00289-z.

Contributions to the literature

- Investigates the facilitators and barriers to the implementation of evidence-based task-strengthening strategies for hypertension control within HIV clinics in Nigeria.
- Identifies strengths and opportunities to leverage within HIV clinics and some challenges to address to optimize for the implementation of task-strengthening strategies for hypertension control within HIV clinics in Nigeria.
- Findings will inform a practice facilitation strategy to enhance the implementation and integration of evidence-based task-strengthening strategies for hypertension control within HIV clinics in Nigeria.

## Introduction

Hypertension (HTN) remains a key risk factor for cardiovascular diseases (CVD), particularly in sub-Saharan African countries currently undergoing the epidemiologic transition of mortality from infectious disease to noncommunicable disease (NCD) [1, 2]. People living with HIV (PLHIV) are at an increased risk for CVD due to an increased prevalence of traditional risk and non-traditional risk factors (i.e., inflammation) as well as the effects of antiretroviral drugs [3]. Evidence-based strategies for HTN control (i.e., World Health Organization’s Package of Essential NCD Interventions for Primary Health Care in low-resource settings (WHO-PEN)) are important but underutilized for hypertension treatment and control among PLHIV [4, 5]. Additionally, the shortage of healthcare workers limits the effective reduction of HTN-related morbidity and mortality rates among PLHIV [6]. Task-strengthening strategies, particularly engaging healthcare workers such as nurses to deliver the WHO-PEN package, may mitigate the sub-optimal HTN control among PLHIV [7, 8]. A cluster randomized control trial in 32 district hospitals and community health centers in Ghana lead by the research team demonstrated that an evidence-based Task-Strengthening Strategy for HTN control (TASSH) based on the WHO Cardiovascular Risk Package led to 1.34 times greater systolic blood pressure reduction than the provision of health insurance [9].

One factor that may impact the integration, implementation, adoption, and sustainability of evidence-based task-strengthening strategies for HTN control is the implementation climate [10]. Defined here as shared perceptions on the importance of evidence-based practice implementation within an organization, Ehrhart et al. [11] suggest that implementation climate creates a fertile organizational context for putting an evidence-based intervention into practice in an organization. The concept has been applied to explore, for example, individual and organizational factors that influence the implementation of interventions for children with autism [12], to optimize clinician’s implementation of evidence-based practice in behavioral health organizations [13], and to assess predictors of engagement in a community-based learning collaborative [14].

Although implementation climate has a robust theoretical foundation [13, 15], few empirical studies have explored its influence on implementing evidence-based practices in sub-Saharan Africa. As a result, closing the gap between research and practice is stymied by the paucity of research on the implementation climate necessary for integrating evidence-based HTN interventions into routine care for PLHIV. Williams et al. [13] suggest that this is an important gap, because once an implementation climate is established, it may potentially have an ongoing, long-term influence on the behavior of healthcare workers, including those who subsequently enter the organizations. There is evidence that practice facilitation, which provides external expertise on practice redesign and promotes a tailored approach to implementing systems changes to improve patient outcomes, may mitigate barriers to implementation climate [13]. It may also generate effective implementation strategies that include healthcare workers’ perceptions and their use of evidence-based practices including what is expected, supported, and rewarded by their organization [11].

In an effort to promote hypertension control among people living with HIV in Nigeria, we are planning for a cluster randomized controlled trial that compares whether practice facilitation (PF) will reduce blood pressure among PLHIV and increase the adoption and sustainability of task-strengthening strategies for hypertension control. The proposed cluster randomized controlled trial would be the first attempt at the integration of HTN management care in HIV clinics in Nigeria. Prior to implementation of TASSH in Nigeria and to maximize the opportunity for success with the integration of evidence-based task-strengthening strategies for hypertension control within HIV clinics in Nigeria, we sought to explore key stakeholders’ current perceptions of barriers and facilitators to the implementation of TASSH in HIV clinics, implementation climate, and organizational readiness for change. Findings will inform the adaptation strategies needed to tailor TASSH implementation to the local context.

## Methods

### Study design and setting

The study was conducted in Lagos, the largest city in Nigeria, and led by the Nigerian Institute of Medical Research (NIMR). We conducted a cross-sectional study design employing an explanatory sequential (Quantitative → Qualitative) mixed-methods approach [16, 17]—a quantitative first step using structured questionnaires followed by a qualitative approach using stakeholder meetings. Step 1 was conducted to quantify implementation climate at the health facilities in relation to TASSH adoption in HIV clinics, followed by step 2—a qualitative focus group discussion during stakeholder’s meeting to explore health providers’ perceptions on the adoption of TASSH at their health facilities and implementation climate. Equal weighting was given to both aspects of this approach. Data were collected between October 2017 and January 2018. Ethical approval for this study was obtained from the NIMR Institutional Review Board. Written informed consent was obtained from the study participant.

### Quantitative identification of HIV clinics and key stakeholders

Quantitative data were collected using a modified practice assessment survey to examine the practice capacity of 29 geographically distinct HIV clinics across 20 different Local Government Areas in Lagos State. The 29 clinics recruited for the study were purposively selected based on the provision of comprehensive antiretroviral therapy (ART) services at the clinic sites and patient load, including private, public/government, faith-based organizations, and primary facility level.

### Qualitative identification of HIV clinics and key stakeholders

For the qualitative aspect, we convened a stakeholder’s meeting of 19 representatives of HIV and noncommunicable disease organizations from Lagos State Ministry of Health, Lagos State Primary Health Care Development Agency, Lagos State AIDS Control Agency, and the Nigerian Institute of Medical research. Stakeholders were purposively recruited to represent individuals who were either directly involved as a participating clinician in the management of NCDs and HIV or involved directly or indirectly at a policy or strategic level.

### Quantitative data collection

We collected quantitative practice assessment data using two instruments: (a) an adapted Service Availability and Readiness Assessment (SARA) tool [18, 19] to assess the capacity of the clinic to manage NCDs and (b) Implementation Climate Scale to assess the degree to which an organization is supportive of evidence-based practice implementation.

#### Service Availability and Readiness Assessment

The SARA questionnaire is a World Health Organization standardized assessment tool to assess the capacity of health facilities to provide basic health services [18, 19]. This tool collects data on essential medicines, technologies, and human resources and has been used in Nigeria [20] and other sub-Saharan African countries [21, 22]. The adapted SARA questionnaire collected data on (1) demographics (provider’s age, educational level, nature of the facility, years of experience in HIV care), (2) organizational characteristics (proportion of HIV patients with a diagnosis of HTN), (3) healthcare provider characteristics (case diagnosis, treatment, referral, and clinic follow-up patterns), and (4) patient characteristics (access to information related to hypertension management and lifestyle behaviors).

#### Implementation Climate Scale

Developed by Ehrhart et al. [11], this modified 14-item scale measures the degree to which evidence-based practices for hypertension management are implemented within the selected HIV clinics. The scale assessed the following: (1) organization focus on EBP for hypertension treatment, (2) educational support for EBP, (3) recognition for EBP, (4) reward for EBP, and (5) openness to implementation. One scale (the selection for EBP) was excluded as it was irrelevant to this context. The objective response options are “2” yes, “1” no, or “0” don’t know, with higher scores indicating a more positive implementation climate.

### Qualitative data collection

We conducted a 1-day in-person stakeholder meeting that lasted for 2 h, hosted by NIMR with the key stakeholders mentioned above. The meeting focus group discussions covered the following: participant’s understanding of the purpose of TASSH and its value in the HIV clinics, their perception of its adaptability and impact on nurses’ work, and suggestions on how they can become engaged in facilitating the adoption of TASSH within the HIV clinics. The meeting was audio-recorded.

### Data analysis

A sequential analytical strategy was applied which involved a quantitative analysis followed by a qualitative analysis. Descriptive statistics (mean and standard deviation, percent or median, and inter-quartile range (IQR)) were used to summarize responses to the survey items using SPSS (Statistical Package for Social Sciences) software version 25. The implementation climate scale measures were examined for internal consistency reliability, and Cronbach alphas were calculated for each subscale. The data from the stakeholder’s meeting (in an audio-recording device) were first transcribed and then analyzed using directed content analysis, which allows for the exploration of phenomenon of interest using a theory as a guide [23]. We used the Consolidated Framework for Implementation Research (CFIR), to thematically analyze data generated from the stakeholder meeting. CFIR provides a pragmatic structure for exploring barriers and facilitators to the implementation of an evidence-based intervention in health systems based on five domains (intervention characteristics, outer setting, inner settings, characteristics of individuals, and the implementation process) [24, 25]. Using a deductive approach, the 5 CFIR domains and 39 constructs were used to identify prior codes to develop the initial codebook. Prior to coding, the research team reviewed and familiarized themselves with the CFIR coding definitions suggested by Damschroder et al. (2009) [25]. Two coders (CO and UN) double-coded the transcript and identified emerging themes related to each CFIR sub-construct and the larger CFIR domain. Discrepancies, such as distinguishing the definitions for intervention complexity and intervention compatibility, between the two coders were resolved by open discussions with a third researcher. The larger research team (JI, CO, UN, DO, AA, and OO) discussed the preliminary themes to reach a consensus on final deductive themes and triangulated responses from the stakeholder’s meeting with survey data. Triangulation was achieved by collecting data from different sources (stakeholders meeting, SARA tool, and Implementation Climate Assessment) to facilitate a deeper understanding of barriers, and facilitators influencing effective implementation from multiple perspectives. We applied Guba’s qualitative trustworthiness criteria to ensure that the data was collected, analyzed, and interpreted accurately [26, 27]. Trustworthiness criteria provide guidelines for qualitative researchers to understand the research context and data (credibility), show consistency and lack of bias in data analysis (confirmability), provide enough detail for possible replication (dependability), and allow for assessment of a study’s outcomes in relation to other contexts (transferability) [27]. Particularly for this study, we maintained an audit trail of the coding process, two authors were involved in the coding process-reading and re-reading the transcribed texts independently in close consultation with the larger researcher team, and discussions and consensus strategies were also used to solve any disagreements among authors during the analysis process.

## Results

### Study participants

The HIV clinics that participated in the survey represented 20 local government areas in Lagos, creating variability in the organizational characteristics and context. The vast majority of the health facilities surveyed were public, government-operated clinics (97%, *n*=28/29). Among the health facility representative surveyed, 62% (*n*=18/29) of the respondents were females, with a median age of 40 years (IQR 37–45 years), and had worked at the current institution for less than 10 years (*n*=21/29). On average, clinic providers care for 394 HIV patients per month. The number of hypertensive HIV patients seen at the facility ranged from 5 to 73 patients per month. Among the participants who attended the stakeholders meeting, 8 of the participants were health providers (noted as HP in the quotes), such as physicians and nurses, within HIV clinics, whereas 11 of them were key informants (noted as SH in the quotes) from the Ministry of Health at the local government level and/or national level.

### Implementation climate for TASSH

As shown in Table 1, most of the scale measures had good–excellent internal reliability in these samples, with Cronbach’s alpha for most subscales ranging from 0.84 to 0.96. The total average score on the organizational context scale was 1.23 (SD=0.46). The focus on the TASSH subscale (1.77 (SD=0.59)) and educational support (1.31 (SD=0.68)) subscale demonstrated the highest mean score. The selection for openness to TASSH and recognition for utilizing TASSH subscales were rated slightly lower at 1.15 (SD=0.73) and 1.14 (SD=0.79), respectively. The rewards for utilizing TASSH subscale were rated the lowest at 0.73 (SD=0.48), which indicates that financial incentives for TASSH use may be uncommon within the clinics.

**Table 1:** Assessment of implementation climate for implementing TASSH within HIV clinics (*n*=29) in Lagos, Nigeria

Implementation Climate Subscales	No. of items	Cronbach's alpha	Mean	SD
Focus on TASSH	3	0.93	1.77	0.59
Educational support for TASSH	3	0.91	1.31	0.68
Recognition for TASSH	3	0.84	1.14	0.79
Rewards for TASSH	2	0.96	0.73	0.48
Selection for openness to TASSH	3	0.88	1.15	0.73
Total	14	0.89	1.23	0.46

### Responses mapped to CFIR

**Fig. 1:**
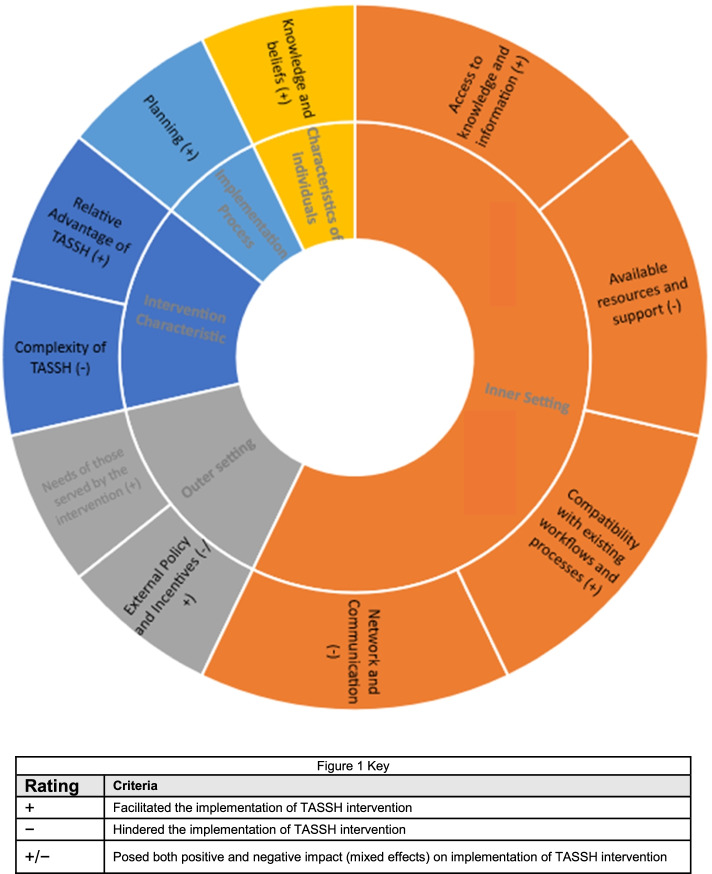
Overview of main influential factors ordered by CFIR domain and constructs

Of the 39 CFIR constructs assessed, 11 of the constructs emerged either as barriers or facilitators of integrating task-strengthening strategies for HTN control within HIV clinics. Figure 1 provides a visual representation of key areas where additional effort or support could be important for successful implementation. Some constructs were found to be more dominant than others based on the degree of importance articulated by the stakeholders and clinic representatives. The CFIR constructs were more concentrated in the inner settings domain compared to the other 4 domains. The relevant constructs within each domain are reported below, including illustrative quotations (Table 2).

**Table 2:** Identified facilitators and barriers based on CFIR domains and constructs

CFIR domain	Barriers/facilitators	CFIR construct	Description
**Intervention Characteristics**	Facilitators	Relative advantage of TASSH	• Reduce the workloads of overburdened workers • Improve overall efficiency • The ability for patients to access care in the same clinic or location • Reduce clinic wait times • Reduce the stigma faced by PLWHIV
	Barriers	Complexity of TASSH	Potential for disagreements and conflicts over roles and role boundaries
**Inner setting**	Facilitators	Compatibility with existing workflows and processes	• Integration of the existing CHEWs into the national structures to improve referral systems
		Access to knowledge and information	• Availability of educational support for evidence-based practice for hypertension management within the HIV clinics
	Barriers	Available resources and support	• Inadequate availability of diagnostic equipment and drugs for HTN across HIV clinics
		Networks and communication	• Weak referral networks and patient tracking mechanism for HTN management within HIV clinics
**Outer setting**	Facilitators	Needs of those served by the intervention	• Supports patients’ needs
		Incentives	• Provision of non-monetary incentives in the form of professional development
	Barriers	External policies	• Existing national polices on NCD management and task-sharing are not implemented in the clinics • No specific action in task-shifting policy to minimize workload
**Characteristics of individuals**	Facilitators	Knowledge and beliefs	• Knowledge of benefit for implementing TASSH and values placed on the importance of EBP
**Implementation process**	Facilitators	Planning	• Accommodate shift preferences and minimize scheduling conflicts for the nurses; Implement simplified data collection tools; align program goals with national guidelines for NCDs

### CFIR-based facilitators with integrating TASSH within HIV clinics

Qualitative themes were generated from the stakeholders’ meetings and quantitative data supporting the findings were generated from the SARA tool.

### The relative advantage of TASSH [CFIR domain: intervention characteristics]

By delegating certain tasks to less specialized health cadres (i.e., nurses), the majority of the stakeholders perceived this model as a solution to make more efficient use of the existing workforce and reduce the workload of overburdened healthcare professionals.

A few health providers highlighted that the rational distribution of the clinical duties among cadres will allow the more specialized healthcare workers to focus solely on clinical tasks and procedures restricted to higher-level cadres. One of the healthcare providers’ notes:

key aspect in the management of hypertension is diet, physical activity, taking appropriate medication and stress management which don’t require the attention of a doctor to achieve…if this task is shifted to them, the Doctors could focus on more complex tasks that require co-morbid management. (HP 1)

Nonetheless, 79% (*n*=23/29) of the HIV clinic representatives surveyed reported that doctors were the sole providers of lifestyle-related information for HTN management such as prompting heart-healthy diet, low sodium intake, physical activity, and eliminating tobacco and caffeine intake.

Another advantage mentioned was the fact that integrating TASSH into routine care for PLHIV will offer patients the ability to access care in the same clinic or location, which may reduce clinic wait times and stigma associated with care-seeking among PLHIV in some settings.

Integrating HIV and Hypertension care would reduce the stigma; it would reduce waiting time and there would be more Doctors available to attend to the patient. (SH 1)

### Compatibility with existing workflows and processes [CFIR domain: inner setting]

Several stakeholders suggested the integration of the existing community health extension workers (CHEWs) into the referral systems, as this may strengthen the compatibility of TASSH intervention within the HIV clinics.

Community Health Extension Workers (CHEWs) could be useful in the tracking of patients, given their close understanding of the community and direct connections with the community members, which could help take some burden off the Nurses. They could serve as a liaison to provide a comprehensive system which starts from the home down to the health system to further strengthen the referral network for the intervention. (SH3)

Within the HIV clinics, less than a quarter of the clinic representatives surveyed indicated that CHEWs and other community health officers are responsible for identifying (21%, *n*=6/29) and referring (14%, *n*=4/29) HIV patients who are hypertensive, suggesting significant gaps in the continuum of care for HTN.

### Access to knowledge and information [CFIR domain: inner setting]

Access to training opportunities and educational resources was perceived as important facilitators for promoting initial uptake and sustained use of new processes within the facilities. This is particularly important as only 52% (*n*=15/29) of health facilities surveyed reported that there were training materials, journals, and other educational resources available for evidence-based practice for hypertension management within the HIV clinics. Similarly, 52% (*n*=15/29) indicated that their facilities provide specific conferences, workshops, or seminars on evidence-based practices. To illustrate the importance of training, one of the stakeholders provided an example where initial training of staff to implement a new process within the clinic gave them a sense of empowerment, thereby increasing their self-efficacy to carry out the given process.

The training allowed them to do things they never imagined they could do and it was realized that research training was important to them as it enhanced implementation across the system and made the Nurses feel empowered. (SH12)

### Needs of those served by the intervention [CFIR domain: outer setting]

Patient needs were a major implementation driver. The majority of the stakeholders supported the goal of the TASSH intervention because they felt that it addressed their long-standing concerns about the growing burden of NCDs among HIV patients at their facilities and how to integrate HTN care into HIV clinics, as described by a stakeholder that,

patients are no longer dying of AIDS at the rapid rate they used to, they are living longer; thus, NCD’s such as cardiovascular diseases and hypertension are now becoming more problematic; the question now is, how can we integrate the treatment of NCD’s within an already existing HIV care. (SH12)

### Incentives [CFIR domain: outer setting]

Stakeholders expressed that health workers at the facilities would expect some form of non-monetary incentives that are both intrinsically and extrinsically motivating to enhance the productivity of health workers with new or additional responsibilities. Some of the suggestions to accommodate the need to incentivize the health workers included social recognition, an increase in professional status, and/or competencies backed by certification.

there is a need to institutionalize the initiative of providing incentives in form of professional development, as Health workers want something to showcase as part of their achievements in their CVs [Curriculum Vitae]. (SH7)

Furthermore, monetary incentives were perceived by the stakeholders as an unsustainable form of compensation for managing health worker’s productivity. All the clinic representatives surveyed (*n*=29/29) indicated that providers at their health facility receive neither financial incentives nor commissions to use evidence-based practices for the management of diseases.

### Knowledge and beliefs [CFIR domain: characteristics of individuals]

Despite the potential of strain that could result from adding on a new responsibility, as highlighted by one of the stakeholders, most of the health providers articulated their belief that the benefits of implementing TASSH within the HIV clinics outweigh any disadvantages. Of the HIV clinic representatives surveyed, 90% (*n*=26/29) of respondents indicated that evidenced-based practices for hypertension treatment are important to the providers in their health facilities.

### Planning [CFIR domain: implementation process]

To avoid the potential of overburdening the health workers, a stakeholder felt that the training required to enable a cadre to take on the new responsibilities should accommodate shift preferences and minimize scheduling conflicts. To do this, it was suggested that the research team consider on-site training at the health facilities as compared to offsite training in the different locations. Other suggestions that emerged within this theme were the need to implement simplified data collection tools to ease adaptation to routine data collection at the clinic and accommodate various reading comprehension levels among the participants. It was also important to the stakeholders that the programs’ goals align with the existing national guidelines for NCDs and clinic activities.

It is imperative to conform, from the beginning of the project to the existing National guidelines for NCDs so that whatever is used will be in line with the priority actions in the guideline. (SH12)

### CFIR-based barriers with integrating TASSH within HIV clinics

#### Complexity of TASSH [CFIR domain: intervention characteristics]

Discussion on the perceived difficulties of implementing the TASSH intervention within HIV clinics centered on challenges with dynamic role boundaries. Many cited the potential for disagreements and conflicts over roles and role boundaries among the cadres of health workers. The issues of power and authority were commonly cited as important factors that may influence relationships and patterns of collaboration among the healthcare teams. In this regard, some of the stakeholders emphasized the need to first understand the scope of practice for the lower-cadre healthcare workers at various facility levels, identify overlapping responsibilities, and then define the roles of the healthcare workers.

We need to look at how services are delivered by these health workers at the primary and secondary care level especially as most patients in HIV care go to the primary care level. (SH7)

persons involved need to know their limitations particularly as Nurses and CHEW are on the same salary scale and have different entry qualifications... We need to clearly define tasks that would be reallocated and let limitations be clearly identified. (SH 5)

### Available resources and support [CFIR domain: inner setting]

Generally, it was found that primary health facilities were supported by the state ministry of health to ensure the availability of basic supplies, diagnostic equipment, and first-line drug regimens related to HIV and hypertension management, but this level of support was not uniform across the different clinics, particularly at lower-level facilities. For instance, only 59% (*n*=17/29) of the HIV clinics surveyed reported that antihypertensive drugs were often readily available at their clinics. This generated some concerns among the stakeholders as they emphatically stated that this deficiency may stifle the implementation of the TASSH intervention within HIV clinics, as resource availability was viewed to be critical in determining the level of staffs’ readiness.

there is a gap, a big discrepancy within the system. Within a small locality, there may be a flagship clinic that is better equipped than a general hospital and within that small locality there may be a primary health clinic that has just one Health care worker (HCW) catering to a lot of people. (SH6)

A select number of flagship clinics are well stocked with drugs and equipment compared to the others. Some buoyant LGA’s have large laboratory which serves the PHC, while at some PHCs, there may have no drugs for the treatment of Hypertension. (SH5)

### Networks and communication [CFIR domain: inner setting]

In relation to referral channels and communications, most of the participants emphasized the need to strengthen referral networks for HTN management in order to support the decentralization of service delivery in the context of the task-strengthening approach. This was seen to be particularly useful in the event that the health worker is faced with patient needs beyond their level of competence which may require higher-level consultation or referral. It was also noted that there were “no proper patient tracking mechanisms” (SH7) in place for intra-clinic referrals and referrals between clinics and hospitals.

### External policies [CFIR domain: outer setting]

In the context of national policies, the national Multi-Sectoral Action Plan for the Prevention and Control of Noncommunicable Diseases (2019–2025) from the Federal Ministry of Health in Nigeria identifies task-strengthening as a priority action for NCD management at all levels of care. In addition, we found that there is an existing task-strengthening and task-sharing national policy for essential healthcare services in Nigeria published in 2014. Although these policies exist, stakeholders highlighted that the policies and guidelines are not implemented at the clinics. Additionally, a stakeholder added that the task-strengthening and task-sharing national policy lacked specific actions for minimizing the workload of health workers in the clinics.

The issue we have now is that there is no policy action that says we can only have a health worker take care of 50 people max, and beyond 50 people no more. (SH12)

## Discussion

This study aimed to examine stakeholder’s perceptions of the implementation climate and other contextual factors necessary for integrating evidence-based task-strengthening strategies for hypertension control within HIV clinics in Nigeria. Using CFIR allowed for a more robust formative assessment that led to the identification of adaptation strategies needed to tailor TASSH implementation to the local context. Understanding the implementation climate from key stakeholders has been identified as a fundamental component for intervention adoption and sustainment, as engaged stakeholders are associated with higher overall positive attitudes towards the adoption of evidence-based practices in their organizations [28, 29]. The results of the implementation climate subscale on the focus of TASSH and the provision of educational support were high indicating a supportive climate for integrating the intervention within HIV clinics in Nigeria. When key stakeholders’ perceptions of TASSH implementation climate, vis a vis a focus on the intervention and provision of educational resources, are high, it signals a shared belief that TASSH may become a lasting priority at the HIV clinics rather than a passing trend.

Indeed, participants were of the opinion that TASSH should be given top priority with managing and controlling high blood pressure rates among people living with HIV. In addition, the provision of in-service training alongside training materials, workshops, and seminars on TASSH were considered to be important for shaping healthcare workers' implementation behavior, ultimately enhancing blood pressure outcomes for people living with HIV. On the other hand, the results of the rewards subscales demonstrated that incentives such as monetary rewards were less likely to affect the implementation of TASSH within HIV clinics. Rather, support and recognitions beyond financial rewards are more likely to grow intrinsic motivation to learn about and adopt TASSH. Taken together, findings illustrate how organizational leaders at HIV clinics can align organizational policies, procedures, and practices and provide educational support to create an implementation climate that shifts healthcare workers’ attitudes and motivations towards the effective use of TASSH in practice. The presence of committed and well-informed organizational leadership and program champions has been identified as key facilitators in other studies that can mitigate organization barriers by maintaining strategic direction and ensuring that organizational resources support intervention adoption [30–33].

In addition to the implementation climate, the results of this study indicate that there are important contextual factors within the organizations that may facilitate or hinder the integration of TASSH within HIV clinics in Nigeria. This finding is corroborated by previous studies that emphasize the importance of the fit between the intervention model and organizational characteristics fundamental for the successful implementation of EBPs [25, 34]. Successful adoption of new evidence-based practices into healthcare settings has been characterized by organizational factors, including adequacy of resources (e.g., training, staffing, and financial) [33, 35, 36]. Specifically, study participants stated that limited resources may hinder the compatibility of TASSH with existing workflows and access to educational support for basic supplies, diagnostic equipment, and first-line drug regimens related to HIV and hypertension management, in addition to the weak referral networks, which underscore the mutability of inner context factors and their potential role in shaping TASSH integration within HIV clinics. Overall, the findings underline the potential role of organizational resources and workforce processes in the successful integration of TASSH within participating study sites. Therefore, implementation of TASSH within HIV clinics in Nigeria would benefit from expert consultation and conducting educational meetings and outreach visits at implementing clinics to enhance intervention-settings fit. The importance of such supportive strategies is consistent with literature on intervention implementation, in which training and education-based strategies are emphasized for implementation success and fit [33].

Likewise, while monetary incentives were described as least beneficial with TASSH implementation climate, some stakeholders believed extrinsic incentives such as organizational recognitions, increase in professional status, and/or competencies backed by certification not only fosters more commitment but gives healthcare workers a sense of ownership with TASSH implementation**.** The relative advantage of TASSH particularly with reducing workloads of overburdened healthcare workers, reducing clinic wait times, and increasing access to care alongside the potential complexity of TASSH underscores how intervention characteristics may exert positive, negative, or potentially neutral influence on the implementation and integration of TASSH within HIV clinics. More research is needed to understand this relationship and to test the impact of healthcare provider-focused interventions on the implementation climate and long-term sustainment of the intervention. However, planning and engagement with key stakeholders throughout the process of integrating TASSH within HIV clinics is an important implementation strategy to promote adoption, fidelity, and sustained use [33, 37].

### Implications for practice facilitation and research

The current study contributes to the existing literature by examining stakeholders’ perspectives of the implementation process and generates new evidence by understanding the implementation climate for the integration of task-strengthening strategies to improve HTN control among people living with HIV. This would provide information to build our understanding of which factors matter and can be used to refine implementation strategies to promote a more wide integration of HTN control into HIV care services and contribute to the field of implementation research [38]. Findings from this study also provide important insights for developing implementation strategies to develop a positive and strong implementation climate within HIV clinics where TASSH will be implemented, such as in the proposed current cluster randomized controlled trial. This study underscores the importance of facilitators training and striving for context fit (e.g., leadership support and availability of resources) to facilitate climates conducive for intervention implementation. Future research is needed to expand our results on how organizational climate drives practice facilitation for evidence-based practices in Nigeria.

### Study strengths and limitations

The results of this study should be interpreted considering some limitations. First, our study is limited to participating organizations and their staff. As such, our findings may not be generalizable to other health organizations. Nonetheless, the organizations selected for the study are within the NIMR network, where the proposed randomized controlled trial will be conducted. Lessons learned from the proposed study could inform implementation in other locations across Nigeria and are transferable to future practice-based interventions. Second, the study excluded other stakeholders, such as the patients, non-governmental organizations, and civil societies, who may have also played an important role in the implementation of the intervention. Third, the study relied on self-reported assessments from the study participants and therefore depends on the stakeholders’ perceptions and relationships with the organizations. While the self-report by stakeholders is an optimal way to measure their perceptions of the organizational climate, they may not necessarily provide an objective evaluation of the organization’s realities or practice capacities of the health centers. Fourth, given the cross-sectional data collection approach utilized for this study, it precludes our ability to make causal statements about the organizational climate that may influence attitudes towards TASSH over time [39]. Future studies should address this limitation by observing trends in perceptions over time to provide a more robust understanding of the implementation climates at the organizations. This can inform the adoption of implementation strategies to best fit the dominant implementation climate.

Despite these limitations, there are several strengths to this study. First, this study utilized both quantitative and qualitative data to improve our understanding of the implementation climate for the integration of TASSH in HIV clinics. The qualitative data corroborated the quantitative findings and added more nuanced information on the implementation climate for TASSH integration [40]. Previous implementation science studies have highlighted the importance of utilizing a mixed-methods approach to achieve a holistic understanding of the implementation’s multiple factors [40–42]. Second, this study included stakeholders from multiple organizational levels, including health providers, policymakers, program coordinators, and program supervisors. Third, to the best of our knowledge, this is the first study to examine the implementation climate for integrating evidence-based task-strengthening strategies for hypertension control within HIV clinics in Nigeria. The knowledge gained from this study will strengthen the implementation of evidence-based practices for HTN control in HIV clinics by offering insights into the barriers and facilitators of successful integration of task-strengthening strategies for HTN control interventions in HIV clinics in Nigeria. Fourth, we utilized a well-established and rigorous implementation science determinant framework, CFIR [25, 43], to frame potential barriers and facilitators for integrating evidence-based task-strengthening strategies for hypertension control within HIV clinics in Nigeria.

## Conclusions

This study provides important insights into barriers and facilitators for implementing evidence-based intervention task-strengthening strategies for hypertension control (TASSH) within HIV clinics in Nigeria. The findings support stakeholder engagement’s importance in fostering collaboration and examining organization readiness for intervention implementation [44]. Organizational readiness is an important factor to understand the implementation climate. Interventions that do not consider contextual and individual factors likely to facilitate or hinder intervention implementation may result in substandard service delivery, compromised health outcomes, and decreased public health impact [45]. Evaluating these contextual factors will lead to increased adoption, implementation, and sustainment of evidence-based intervention and an overall improvement in public health impact. Future research should focus on identifying strategies to support the long-term practice sustainment of TASSH in Nigeria. Such approaches hold promise to improve the implementation and sustainment of intervention, as well as the quality and outcomes of care.

## Supplementary Information

**Additional file 1.**

## Data Availability

Data is available upon request to the corresponding author

## Abbreviations

AA: Angela Aifah
AIDS: Acquired immunodeficiency syndrome
AM: Adesola Z. Musa
AR: Ashlin Rakhra
ART: Antiretroviral therapy
BT: Bamidele O. Tayo
CFIR: Consolidated Framework for Implementation Research
CHEWS: Community healthcare workers
CO: Chisom Obiezu-Umeh
CVDs: Cardiovascular diseases
DO: David Oladele
DO^2^: Deborah Onakomaiya
GO: Gbenga Ogedegbe
HCW: Healthcare worker
HIV: Human immunodeficiency viruses
HJ: Hu Jiyuan
HP: Health providers
HTN: Hypertension
II: Ifeoma Idigbe
IQR: Interquartile Range
JG: Joyce Gyamfi
JI: Juliet Iwelunmor
LGA: Local Government Area
NCDs: Noncommunicable dseases
NIMR: Nigerian Institute of Medical Research
OE: Oliver Ezechi
OO: Oluwatosin Odubela
PF: Practice facilitation
PHC: Primary health care
PLHIV: People living with HIV
SARA: Service Availability and Readiness Assessment
SD: Standard deviations
SPSS: Statistical Package for Social Sciences
TASSH: Task-strengthening strategies for hypertension
TG: Titilola Gbajabiamila
UN: Ucheoma Nwaozuru
